# Major Low Anterior Resection Syndrome (LARS) and Quality of Life in Patients With Low Rectal Cancer: A Preoperative Survey Using LARS Score and European Organisation for Research and Treatment of Cancer’s 30-Item Core Quality of Life Questionnaire

**DOI:** 10.7759/cureus.50074

**Published:** 2023-12-06

**Authors:** Ly Huu Phu, Ho Tat Bang, Nguyen Viet Binh, Hoang Danh Tan, Ung Van Viet, Nguyen Trung Tin

**Affiliations:** 1 Gastro-intestinal Surgery, University Medical Center HCMC, University of Medicine and Pharmacy at Ho Chi Minh City, Ho Chi Minh City, VNM; 2 Thoracic and Vascular Department, University Medical Center HCMC, University of Medicine and Pharmacy at Ho Chi Minh City, Ho Chi Minh City, VNM; 3 Health Organization and Management Department, Faculty of Public Health, University of Medicine and Pharmacy at Ho Chi Minh City, Ho Chi Minh, VNM; 4 Proctology Department, University Medical Center HCMC, University of Medicine and Pharmacy at Ho Chi Minh City, Ho Chi Minh City, VNM; 5 General Surgery, Faculty of Medicine, University of Medicine and Pharmacy at Ho Chi Minh City, Ho Chi Minh City, VNM; 6 General Surgery, Faculty of Medicine, University of Medicine and Pharmacy at Ho Chi Minh City, Ho chi Minh City, VNM

**Keywords:** vietnam, lars score, eortc qlq-c30, preoperation, quality of life, low anterior resection syndrome

## Abstract

Background

Rectal resection with total mesorectal excision is a difficult surgery with potential risks of complications. This study aims to assess the quality of life (QoL) of patients with low rectal cancer who have bowel function disorders equivalent to major low anterior resection syndrome (LARS) and its risk factors before treatment.

Methods

A descriptive cross-sectional study was conducted on 83 patients diagnosed with low rectal cancer who had not been treated. Quality of life was assessed by the European Organisation for Research and Treatment of Cancer's (EORTC) 30-Item Core Quality of Life Questionnaire (QLQ-C30) and the LARS scale.

Results

Fiffty-five (66.3%) patients had moderate/major low anterior resection syndrome, of which 34 (41%) patients had major low anterior resection syndrome. The study implicated that old age, smoking, and alcohol consumption were risk factors associated with high scores on the scale for LARS (p<0.05). Patients with low rectal cancer had low overall QoL score. In the symptom area of increased financial hardship scores, factors that adversely affected the poor quality of life in patients with low rectal cancer were fatigue and bowel dysfunction with p<0.05.

Conclusion

The percentage of rectal cancer patients with low anterior resection syndrome was high, and the associated risk factors were old age, smoking, and drinking alcohol. Before treatment, the physical and mental health of patients with low rectal cancer with major low anterior resection syndrome was very poor.

## Introduction

The treatment of low rectal cancer is a multimodal approach consisting of low anterior resection with total mesorectal excision (TME) combined with adjuvant chemotherapy and radiation therapy. Rectal resection with total mesorectal excision is a challenging surgery with a risk of complications such as presacral bleeding, anastomotic leaks, rectovaginal fistula, and residual abscess. Complications caused by surgery or side effects of chemotherapy and radiotherapy have greatly affected the physical and mental health of the patient [1-4]. Many studies show that patients' quality of life (QoL) after rectal resection has severely deteriorated [5,6]. Symptoms of bowel dysfunction (also known as low anterior syndrome) include obstructed defecation and incomplete evacuation of the stool. These symptoms are common and severely impact the patient’s QoL. In some cases, they persist throughout the patient’s life and can be troublesome. Some patients cannot tolerate the condition and undergo surgery for a permanent artificial anus [7,8].

Many recent studies show that providing patients with information about the progression of their disease, along with information about potential complications or sequelae associated with the treatment, may guide medical staff in planning optimal, comprehensive treatment and assist patients in choosing a treatment method that suits their circumstances [9]. Thereby, it helps to increase the effectiveness of treatment while improving the patient’s QoL. There are few current studies on this issue, and most of them are retrospective.

This study aims to establish how many patients with low rectal cancer experience severe bowel dysfunction symptoms and how it impacts their QoL-concurrently, aiming to identify the risk factors for severe bowel disorders. By gathering this information, healthcare workers can plan treatment to improve the physical and mental health of patients, ultimately enhancing the quality of care they receive.

## Materials and methods

Objective

To determine the proportion of patients with low rectal cancer who have severe bowel dysfunction equivalent to major low anterior resection syndrome (LARS) and its effect on their before-treatment quality of life (QoL).

Study settings and participants

A cross-sectional study was conducted on patients aged 18 years or older, diagnosed with low rectal cancer, and who have not been treated at University Medical Center, Ho Chi Minh City between March 2022 and March 2023. The patients agreed to participate and had adequate cognitive ability to answer two questionnaires.

Data collection and tools

Data was collected by conducting interviews and referring to the medical records of the gastrointestinal and rectal surgery departments of the University Medical Center, Ho Chi Minh City. A treatment plan for low rectal cancer was decided by the multimodal consultation panel when the patient was diagnosed. A researcher introduced the patient to the study, if the patient agreed to participate, the researcher would provide them with two multiple-choice questionnaires. Once completed, the researcher verified that the patient had fully answered the questions.

The two questionnaires collected data about the patient’s social, clinical, and QoL variables. The first questionnaire was the European Organisation for Research and Treatment of Cancer's (EORTC) 30-Item Core Quality of Life Questionnaire (QLQ-C30) questionnaire, which assesses the general QoL for all cancer patients. The second questionnaire was the LARS score, which assesses the symptoms of bowel dysfunction and its impact on the patient’s QoL. This study used the EORTC QLQ-C30 version 3.0 questionnaire, which has been translated into Vietnamese (released and applied since 2000) and has been used in many different research projects to assess the general QoL of cancer patients [10]. There are 30 questions, five functional scales, three symptom scales, one general health condition, and six individual symptoms. The EORTC-C30 questionnaire was designed to assess the QoL across multiple aspects, such as physical activity, cognitive ability, social integration, emotions, and systemic symptoms caused by the disease or by cancer treatment. For each factor, the average score of the component statements in the scale will be reported.

The LARS score questionnaire was released in 2012 and has been proven to be of reliable scientific value for assessing bowel dysfunction and its effects on the QoL of patients following rectal resection [11]. The LARS score consists of five questions relating to the five main symptoms of LARS. Bowel dysfunction is divided into three levels of severity: major (30-42 points), moderate (21-29 points), and no LARS (0-20 points) to assess its impact on each patient’s QoL. The LARS score questionnaire has been validated in Vietnam, with the test-retest reliability of 89 patients showing a high intraclass correlation coefficient [12].

Statistical analysis

The research data were entered using Epidata 4.6 (EpiData Classic, Data Management and Basic Statistical Analysis System. Odense Denmark) and analyzed using SPSS (IBM Corp. Released 2011. IBM SPSS Statistics for Windows, Version 20.0. Armonk, NY: IBM Corp). One-way analysis of variance (ANOVA) and Fisher’s exact test were employed to measure significance associations, p<0.05 when the QoL scores were normally distributed. We use the Kruskal-Wallis test and the Spearman correlation to analyze cases where the QoL scores exhibited an abnormal distribution.

Research ethics

This work was conducted under the approval of the Ethics Committee in Biomedical Research at the University of Medicine and Pharmacy at Ho Chi Minh City, granted on March 10, 2022 (Decision No. 295/HĐĐĐ-ĐHYD).

## Results

Eighty-three patients were included in the study. Demographic characteristics and clinical examinations are described in Table 1. There were more males than females, with 54% males included (45 patients). The mean age was 61.5 ± 11.2, ranging from 25 to 84 years old. Fifty-four patients (65.1%) lived in urban areas. Almost all patients had cancer at an advanced stage (77 patients or 92.8%), and only seven patients (7.2%) had been diagnosed at an early stage. Almost 25% of all patients had neo-adjuvant chemoradiotherapy before surgery. The study shows that 58 patients (almost 70%) had a good health status [American Society of Anesthesiologists (ASA) grade I or II]. A tumor height of 6-10 cm was found in 52 patients (62.7%).

**Table 1:** Sample population demographics a mean±standard deviation; b interquartile range; *distance from anal verge; ASA: American Society of Anesthesiologists

Characteristics	Frequency	Percentage (%)
Age	61.5 ± 11.2^a^ (25–84)^b^	
Gender		
Male	45	54.2
Female	38	45.8
Living place		
Urban	54	65.1
Rural	29	34.9
Academic level		
Elementary	34	41.0
High school	36	43.3
University	13	15.7
Career		
Manual labor	28	33.7
Mental labor	10	12.1
Aged, retired	45	54.2
ASA grade		
Grade I	9	10.9
Grade II	49	59.0
Grade III	25	30.1
TNM stage		
1^st^ stage	6	7.2
2^nd^ stage	8	9.7
3^rd^ stage	67	80.7
4^th^ stage	2	2.4
Tumor height*		
5cm	31	37.3
6–10cm	52	62.7

Fifty-five patients (66.3%) noted bowel dysfunction symptoms that were classified as moderate to major LARS. Thirty-seven patients (66.1%) with middle rectal tumors had LARS symptoms, while 18 patients (66.6% ) with lower rectal tumors reported LARS symptoms. The proportion of patients with major LARS was 41% (34 patients). Those with middle and lower rectal tumors and major LARS were 37.5% (21 patients) and 48.1% (13 patients), respectively (Table 2).

**Table 2:** The ratio of patients with symptoms related to LARS LARS: low anterior resection syndrome

	Entire rectum (N,%)	Mid rectum (N,%)	Low rectum (N,%)
Low anterior resection syndrome			
No LARS	28 (33.7)	19 (33.9)	9 (33.3)
Moderate LARS	21 (25.3)	16 (28.6)	5 (18.5)
Major LARS	34 (41.0)	21 (37.5)	13 (48.1)
Total (N,%)	83 (100.0)	56 (100.0)	27 (100.0)

Advanced age and age group division were associated with the severity of LARS, and the difference was statistically significant (p=0.031). There was an association between the severity of low anterior resection syndrome and the stage of the disease; the difference was statistically significant (p-value was 0.023). There was no association between low anterior resection syndrome and risk factors such as gender, chronic disease, ASA, body mass index (BMI), or tumor location. The degree of invasion of the tumor (Table 3).

**Table 3:** Factors related to LARS a mean±standard deviation; #Frequency (percentage) LARS: low anterior resection syndrome; ASA: American Society of Anesthesiologists (ASA) physical status classification system; cTNM: Clinical Tumor – Node – Metastasis stage; BMI: body mass index

	No LARS	Moderate LARS	Major LARS (N=34)	p value
	(N=28)	(N=21)		
Age (year)^a^	59.4 ± 12.6	58.6 ± 11.1	64.9 ± 9.4	0.047
Age group ^#^				0.031
<60	14 (50.0)	10 (47.6)	7 (10.6)	
≥60	14 (50.0)	11 (52.4)	27 (79.4)	
Gender^#^				0.524
Male	14 (50.0)	10 (47.6)	21 (61.8)	
Female	14 (50.0)	11 (52.4)	13 (38.2)	
BMI (kg/m2)^ a^	21.9 ± 2.4	21.7 ± 2.5	21.2 ± 2.9	0.343
ASA grade^#^				0.645
I	5 (17.9)	2 (9.5)	2 (5.9)	
II	16 (57.1)	13 (61.9)	20 (58.8)	
III	7 (25.0)	6 (28.6)	12 (35.3)	
Chronic disease^#^	11 (39.3)	12 (57.1)	16 (47.1)	0.481
Tumour location^#^				0.556
Middle rectum	19 (67.9)	16 (76.2)	21 (61.8)	
Lower rectum	9 (32.1)	5 (23.8)	13 (38.2)	
Smoking	4 (14.3)	2 (9.5)	13 (38.2)	0.02
Drinking alcohol^#^	4 (14.3)	0 (0.0)	12 (35.3)	0.003
cTNM before surgery^#^				0.023
I, II	9 (32.1)	3 (14.3)	2 (5.9)	
III, IV	19 (67.9)	18 (85.7)	32 (94.1)	
Tumor in vasion^#^				0.138
T1,T2	6 (21.4)	1 (4.8)	2 (5.9)	
T3,T4	22 (78.6)	20 (95.2)	32 (94.1)	

Before treatment, the QoL score in terms of physical activity in all three groups of low anterior resection syndrome was normal. The overall quality of life score were poor in all three groups, higher in the no LARS group (50.6 ± 9.3), and lower in the others. The difference was statistically significant (p=0.004). In terms of fatigue, the average score increases as LARS levels rise. Specifically, the no LARS group showed the lowest average score (12.7), the moderate LARS group showed an average score of 17.5, and the major LARS indicated the highest average score of 22.9 (p=0.041). There was a statistically significant difference between digestive disorder average scores and LARS levels (p=0.003). The lowest score was found in the LARS group (6.0). The highest was shown in the moderate group (15.2) (Table 4).

**Table 4:** QoL score according to EORTC C-30 with LARS a mean±standard deviation

Domain	No LARS (N=28)^a^	Moderate LARS (N=21)^ a^	Major LARS (N=34)^ a^	p-value
Physical activity	94.3 ± 9.6	91.4 ± 12.1	88.6 ± 20.0	0.425
Social role	85.1 ± 23.7	81.0 ± 27.5	77.0 ± 22.5	0.145
Social intergration	51.8 ± 26.2	42.9 ± 22.1	51.5 ± 18.1	0.345
Psychology, emotion	53.6 ± 19.6	48.4 ± 15.9	55.1 ± 24.2	0.314
Cognitive ability	92.3 ± 14.7	92.9 ± 14.5	89.2 ± 15.8	0.465
Overall QoL	50.6 ± 9.3	44.0 ± 10.9	44.1 ± 10.0	0.004
Fatigue	12.7 ± 19.5	17.5 ± 18.8	22.9 ± 18.3	0.041
Pain	7.1 ± 16.0	15.1 ± 18.2	14.7 ± 18.7	0.106
Insomnia	19.0 ± 21.1	23.8 ± 23.9	23.5 ± 25.3	0.766
Breathing difficulty	4.8 ± 11.9	7.9 ± 18.0	5.9 ± 15.3	0.865
Digestive disorder	6.0 ± 7.6	15.2 ± 13.2	13.9 ± 10.0	0.003
Financial difficulty	38.1 ± 33.6	42.9 ± 31.9	26.5 ± 22.9	0.145

## Discussion

The study showed that before treatment, the QoL of rectal cancer patients was low in functional aspects such as social integration, psychological-emotional state, and social roles. The overall QoL was heavily affected. In patients with bowel dysfunction, QoL was further reduced. Research by Moseholm et al., Kwoun et al. and Kim et al. supported this finding [6,13,14]. Another noted that patients with psychological disorders who had poor bowel dysfunction were at a higher risk of complications and bowel dysfunction after surgery and were at risk of being re-admitted to the hospital [15].

In addition, in terms of symptoms, we found that fatigue and insomnia had a negative impact on the patient’s QoL. Research by Kim et al., Qedair et al., and Pieniowski et al. supports this finding [14,16,17]. The results showed that financial difficulty is one of the factors correlated with a patient’s QoL score. Most of the patients in this study were manual workers on low incomes. Low financial resources can lead to limitations to the patient’s treatment options, especially after the COVID-19 pandemic.

Similarly, evidence showed that financial difficulties were a huge burden that adversely affected the physical and mental health of rectal cancer patients in Southeast Asian countries, especially those on low incomes, leading to less effective treatments [18]. Recently, the results of some studies have shown that the more concerning issue was the progression of LARS, which was prolonged and persistent, affecting the patient’s QoL for the rest of their life. Dinnewitzer et al. noted that 20% of patients could not tolerate it, choosing to undergo further surgery to have a permanent artificial anus in the hope of a better QoL [8]. Therefore, healthcare workers should evaluate and classify patients by risk group before treatment, which would help reduce the number of patients with severe and persistent low rectal syndrome later.

Our study shows that advanced age is a risk factor associated with major LARS. The group of older patients had more severe symptoms of bowel dysfunction than the group of younger patients; this finding is supported by earlier studies [14,17,19].

Two-thirds (66.3%) of patients in the study had bowel dysfunction equivalent to LARS, 41% of patients diagnosed with severe symptoms. The proportion of men and women with major LARS was 46.7% and 32.4%, respectively. This bowel dysfunction may be caused by tumors or impaired physiological function in the anorectal sphincter that occurs in patients of advanced age, or both. In 2018, Juul et al. studied 1,875 Danes, providing us with important data. In the population of 50-75-year-olds, the proportion of men and women with symptoms of bowel dysfunction equivalent to major LARS unrelated to disease or rectal surgery was 9.6% and 18.8%, respectively [20]. Our study indicates that advanced age is a risk factor for major LARS, which surgeons should consider carefully before performing sphincter-conserving surgery in older patients.

Before treatment, we noted that fewer men had rectal cancer than women, yet a higher proportion of men had severe LARS compared to women. These results are similar to the results by Kim et al. and Cheong et al.. However, the difference in our study was not significant. The study by Kim et al. and Cheong et al. showed that males were at a greater risk of major LARS at the time of surgery [14,21].

According to Alavi et al., low rectal tumors (≤6cm from the anal margin) and smoking were risk factors associated with major LARS [22]. In 2018, the pre-operative LARS score (POLARs) study was conducted by Battersby et al. in Anglo-Denmark to identify the predictive risk factors for patients with major LARS after low anterior resection, including advanced age, total mesorectal excision, neo-adjuvant radiotherapy, tumor location to anal margin ≤6cm, and temporary artificial anus condition [23]. Our study shows some similar factors. In addition, we also recognized that drinking alcohol was a risk factor associated with severe bowel dysfunction.

This study was one of the very few prospective studies evaluating the QoL of patients with low rectal cancer before treatment. The study provided the proportion of patients with severe bowel dysfunction and the QoL of patients at the time before treatment. In addition, it also showed some predictive risk factors. It helps clinical practitioners make better decisions for their patients based on evidence.

There are some limitations. Firstly, a cross-sectional study collected data on 83 patients. The small sample size may lead to bias when exploring the related factors. However, we tried to use appropriate statistical methods to reduce its effect. Additionally, a cohort study with a control group is needed to identify the risk factors clearly.

## Conclusions

The percentage of rectal cancer patients with low anterior resection syndrome was high, and the associated risk factors were old age, smoking, and drinking alcohol. Before treatment, the physical and mental health of patients with low rectal cancer with major low anterior resection syndrome was very poor. Therefore, comprehensive assessment and a supportive plan for patients were necessary before surgery, helping to improve treatment efficiency and quality of life.
